# Reflections on trust and COVID-19: do politics, medicine and the environment need each other?

**DOI:** 10.14324/111.444/ucloe.000010

**Published:** 2020-08-27

**Authors:** Alistair Cole, Frederic Dutheil, Julien S. Baker

**Affiliations:** 1 Department of Government and International Studies, Hong Kong Baptist University, Kowloon Tong, Hong Kong; 2 Preventive and Occupational Medicine, University Hospital of Clermont-Ferrand (CHU), Clermont-Ferrand, France; 3 Department of Sport, Physical Education and Health, Hong Kong Baptist University, Kowloon Tong, Hong Kong

**Keywords:** trust, environment, medical and social sciences, international comparison, transdisciplinarity, COVID-19, political science, policy and law

## Abstract

This short article is centred on how trust can be a valuable resource for developing cognate responses to the COVID-19 pandemic in the medical and social sciences. Politics and medicine can learn from each other. Governments need to persuade individuals to adapt their behaviours, and such persuasion will be all the more convincing in that it is nested in social networks. Trust in government requires consistent (benevolent, performative and joined-up) explanations. The distinction between hard medical and soft social science blurs when patients/citizens are required to be active participants in combatting a pandemic virus.

Scientists and politicians seem sometimes to inhabit very different worlds. The COVID-19 pandemic has provided numerous examples of this. There is, at the least, a politics–science temporal misfit: the true value of science, requiring rigorous testing, predictably makes the search for a vaccine a slow process, one not in the same time frame as that required by the political management of the crisis, which requires ‘fast’ solutions. This special issue is more generally concerned with the social implications of the pandemic and the environmental changes connected to it. Continuing the analysis, the environmental temporality might be interpreted as a long-term tragedy, in the Shakespearean sense of a visible, impending and relentless movement. Or, alternatively, as a more heroic enterprise of apprising the collective consciousness of the necessity to act in time to avoid catastrophe and preserve the global commons. The evidence provided by the COVID pandemic is ambivalent, however, being open to dual readings, so much so that it is unwise to edict clear causal relationships.

There are overwhelming common interests in agreeing on the terms of reference. From a comparative politics perspective, COVID-19 provides a vast living dataset to engage in multi-level comparisons and real-time experiments. In the medical research field, the pandemic has provided advances in medical science that would not have been possible without access to a living laboratory. Science has occupied centre stage and forms part of the struggle of the narratives between countries and regions. From liberal democratic countries such as the UK and France to semi-authoritarian regimes such as Singapore, governments have justified decisions based on the ‘scientific evidence’. Politicians in many countries involve medical experts in the announcement of infections, deaths and policy measures to combat the pandemic. Medical experts appear more trustworthy than politicians and their pronunciations are taken more seriously by the general public. Trust in health professionals is the core finding across these very different types of political systems. One interesting angle relates to the role of these professionals. Are they co-constructors of public policy? In countries such as the UK and France, the governments have created scientific advisory committees and justified their decisions based on the ‘scientific evidence’. Or are the health professionals there mainly to provide scientific caution? In the mentioned examples, and more generally, governments have sought to avoid blame by co-opting scientists. On occasion, medical practitioners emerge as the gatekeepers of public health, as in the case of the doctors’ strike in Hong Kong forcing the government there to close the border with China. These are key questions. Although they have been harshly criticised, democratic governments, such as that of the UK, are bound by a degree of public transparency, hence they are more likely to admit mistakes than their authoritarian counterparts.

The COVID-19 pandemic also demonstrates the contested nature of medical knowledge: as testified by the controversies over hydroxychloroquine or contrasting containment strategies (herd immunity vs. lockdown). Let us not forget that, in the case of the UK, the herd immunity approach was the one advocated by leading government scientific advisors. In the absence of an effective global commons, moreover, the crisis has revealed the barriers to the functioning of a global medical epistemic community, free to exchange ideas/information in the interests of scientific discovery. Health professionals have been at the forefront of the fight against COVID-19, but as the case of China in particular suggests, they are not always free to tell the truth [1].

Referring to the global commons provides the best entry point for linking the analysis of COVID-19 with the environment. Of course, deep concern about the future of the planet precedes COVID-19. The Paris climate treaty (COP 21) of 2015 represented a highlight of international collective action to fight global warming. But the decision by the United States (US) administration to withdraw from the Paris agreement (along with withdrawing from a host of other international organisations and agreements) was a harbinger of future trends in the troubled Trump presidency. The COVID-19 crisis – or the Wuhan epidemic as described by Trump – demonstrates the deep mistrust between the key global actors whose cooperation is needed to fight the battle to preserve the planet. Hence, the difficulty of imputing clear causal effects to COVID-19 and its impact on the environment. Rather, paradoxically, the short-term impact of COVID-19 would appear to be beneficial to the environment: CO_2_ emissions were down in all the major urban centres as a result of industrial shutdowns and the lack of travel mobility (whether individual or collective) [2,3]. But the longer term implications of decoupling, one probable consequence of COVID-19, are potentially extremely damaging, even on an existential level.

Responding to existential dilemmas, the COVID-19 pandemic calls for a major transdisciplinary research effort that necessarily combines several levels of empirical analysis and bridges distinct academic and scientific traditions. This short article is centred around the question of whether COVID-19 represents, inter alia, a crisis of trust, one theme that successfully links the social and medical sciences. Health scandals, such as mad cow disease (bovine spongiform encephalopathy, or BSE) in the UK or the tainted blood scandal in France, have a particular place in the interface between citizens and their trust in politics and the health and related professions [4]. Politicians and medical scientists also share common interests, not least in correctly diagnosing health crises and conceptualising the role of trust therein.

Literature from political science, especially relating to the three levels of trust of Zmerli and Newton [5], allows a fairly precise operationalisation. Trust needs to be understood as a generic term to describe the dynamics taking place at different levels of analysis: interpersonal, social and collective. Medical and social science rely on theorising at three main levels of analysis: individual, intermediate and institutional. Each type of analysis carries a distinctive contribution and the stakes of each are high: psychological wellbeing, civil society and trust in government. In medical and political circles, there is an enormous leap from individual-level analysis, through to socially mediated forms of trust and onto the headline events such as the crisis of trust in the US health system identified by Sisk and Baker [6]. In terms of comprehension, trust does not necessarily gain from moving between these levels of analysis in an indiscriminate manner, but substantive distinctions are important.

*Inter-personal trust* provides the first level of analysis. In their Trust, Confidence and Cooperation model, Earle and Siegrist [7] distinguish between trust and confidence. Trust is defined as the willingness to make oneself vulnerable to another based on a judgement of similarity of intentions or values. It involves an inter-personal relationship, with at least two players, as in a doctor–patient relationship. Confidence is defined as the belief based on past experience or evidence that certain events will occur as expected. Both trust and confidence influence people’s willingness to cooperate. In terms of both trust and confidence, the individual level is key, because individual perceptions of risk are germane to the adoption of preventive measures [8].

In another close formulation ‘particular social trust’ involves those known to us personally, such as family, friends or work colleagues. A breakdown of trust shatters this psychological equilibrium. Cross-national evidence from lockdowns and confinements illuminates the challenged state of psychological wellbeing of individuals, especially in terms of their primary networks (friends, family) and practices (as a result of social distancing). Even within these tight personal networks, evidence from scholars working on psychological indicators points to an increase in indicators of social tension, such as divorce, gender violence and isolation, as a result of the COVID-19 crisis [9]. Increases in social violence and violation by communities in relation to social distancing measures are major concerns in relation to public perceptions and information provided by respective governments and their representatives.

There is an environmental dimension to this. Social capital is literally excluded as a result of lockdown and travel restrictions (creating a lasting impact on the travel industry and on individual mobility patterns, all contributory factors to broader environmental effects). But society has not collapsed. Even where confinement was harsh, as in Italy, Spain and France, the strength of civil ties allowed the crisis to be weathered. The short-term impact of COVID-19 is one of lessening consumption, the counterpart of restraints on freedom and mobility, and a calling into question of the legitimacy of individual-level preferences in terms of mobility. How long such mechanisms can last in market economies remains to be seen. The trust dimensions intervene at two main levels: trust in government and governmental advice and trust in scientific expertise. Effectively confronting a pandemic requires the active collaboration of individuals and civil society.

*Social or collective trust* is the second level of analysis. ‘General social trust’ is that placed in ‘unknown others’. This form of trust performs a key function in modern societies, as Newton [10: 349] notes, because ‘much social interaction is between people who neither know one another nor share a common social background.’ In relation to COVID-19, the ability to empathise with members of an imagined community (region, nation, even continent) is a core element of community integration. How civil society has reacted to the pandemic is a matter of empirical investigation, but society has not collapsed, as was seen in Italy, Spain and France. Public participation is central to the success of adopting preventive measures, including in respecting governmental advice in relation to social distancing, limiting travel and so on. Insofar as COVID-19 limits social interaction and contact, it probably carries with it certain short-term benefits – such as lessening emissions, improved air and water quality. But does it also represent a long-term cognitive shift?

Such an analysis depends on how citizens place their trust in government, our third dimension; this has performed a major role during the pandemic by affecting the public’s judgements about risks and related benefits [8]. From a psychological perspective, Bish and Michie [11] argue that trust in government is a key variable affecting individual behaviour when faced with pandemics; the more consistent the message, the more likely it is to influence behaviour. Misinformation and lack of action in the early stages by governments has led to an apathetical approach by communities and a feeling of identity immunity. Citing studies of various countries, Siegrist and Zingg [12: 25] suggest that ‘trust had a positive impact on adopting precautionary behavior during a pandemic’. Trust in government is important; even more central is trust *and* confidence in experts. During pandemics, most people are not in a position to evaluate the information about the risks and benefits associated with vaccination. Therefore, they rely on experts, especially on those experts they trust, who are once removed from government. This finding was backed by van der Weerd et al. [13] in their study of the H1N1 flu pandemic in the Netherlands. From these various studies, clear consistent ethical guidelines are called for by the medical community [14].

Governmental policy towards individual and collective mobility (transport) has included at least as much ‘fudge’ as* ‘*nudge’. Governments have certainly attempted to persuade (nudge) their populations to exercise social control, sometimes using the threat of fines for transgression. But they have also fudged the issue, sending mixed messages, for instance, in the case of air transport in the UK, where the border was open until June 2020 against most interpretations of sound logic, only to be closed when other countries began to open their own borders. Contrasting responses do not simply relate to types of government. This issue is not a simple one of distinction between types of polity, for example, federal versus unitary systems: while the US and Brazilian federations descended into partisan-based rivalries between states, federal Germany demonstrated one of the most joined-up responses to the pandemic.

At the *international level*, mistrust is one dimension of the ongoing great power rivalry between the US and China, with Europe, in the main, a perplexed and wounded bystander. Controversy over the provenance of the pandemic – and the appropriate description of it – is an intervening variable of the increasingly bitter global contest between China and the US – and Xi Jinping and Trump in particular. Mistrust is inherent in realist framings of the international system, but COVID-19 has acted as a step change in the deterioration of Sino-American relations. The environment might well be one victim of the growing mistrust within global institutions. There is a real danger of ‘decoupling’, of Asia and America inhabiting different spheres of existence. The difficult governance of international organisations – such as the World Health Organization, which the US gave notice of its intention to quit in the midst of the COVID-19 pandemic – stems in part from a suspicion of Chinese influence. There is, in this sense, a direct link between the pandemic and the weakening of multilateral institutions. However, there are also difficulties of interpretation: does COVID-19 symbolically represent a brutal stop to globalisation? Or is it too early to tell?

The level only describes one part of the dynamic. Adapted to medical ethics, for example, practitioner and client relationships span elements of inter-personal and intermediate trust. The appropriate relationship is prescribed and described in medical ethics (with trust as a form of confidential relationship, absolutely secretive, in no sense negotiated with a third party) and regulated by strict professional and ethical standards. Here, the medical relationship is stronger than even the tightest form of inter-personal political relationship. Nonetheless, COVID-19 challenges interpersonal trust in a novel manner; traditional consultative practices are changing (e.g. e-Medicine), while the competition for scarce resources has ensured that COVID-19 eclipses more traditional treatments (e.g. the postponing of cancer operations).

The key analytical point is that, in the medical sphere as in the political one, discussion centres on the linkages between individual, intermediary and organisational levels of analysis. The COVID-19 pandemic has focused attention on the need to strengthen the links between patients, health care teams and organisations. From the relevant literature, we learn that the most effective responses in a pandemic are joined-up ones, where individuals (responsible for following guidelines) trust intermediaries (health professionals) and are receptive to messages (nudges) from the relevant governmental authorities. Hence, the distinction between hard medical and soft social science blurs when patients/citizens are required to be active participants in combatting the virus.

Politics and medicine can learn from each other. Levels of trust are most effective when combined. In terms of trust, individual responses and reactions, social mediation and governmental responses need to complement each other. In a pandemic, governments need to persuade individuals to adapt their behaviours, and such persuasion will be all the more convincing in that it is nested in social networks. Here, reverting to the advice of psychologists and medical scientists assumes its own coherence. Clear and consistent legitimising discourses are, almost by definition, most difficult to sustain in a crisis, the political equivalent of Schumpeter’s creative destruction. However, trust in government requires consistent (benevolent, performative and joined-up) explanations. The evidence drawn from the medical and social science perspectives goes in the same direction.

The three-level analysis is equally appropriate in the field of the environment. Individual level behaviour lies at the very heart of environmental challenge, but this takes place within the bounds of social acceptability – at least in pluralistic, liberal democratic societies. Governments need to provide accessible information, not in terms of information overload, but clear consistent guidelines. Evidence – from Trump, Macron and Johnson, for example – suggests that contradictory information is associated with reduced public trust. Global governance is more than ever necessary to rise to the environmental challenge – but possibly is the main victim of the geo-political consequences of the COVID-19 crisis.

## Data Availability

Data sharing is not applicable to this article as no datasets were generated or analysed during the current study.
